# Survival data on timing of resection of liver metastases in colorectal cancer patients

**DOI:** 10.1016/j.dib.2020.105973

**Published:** 2020-07-03

**Authors:** Ulrich Nitsche, Constance Weber, Benedikt Kaufmann, Guido von Figura, Volker Assfalg, Gregor Miller, Helmut Friess, Norbert Hüser, Daniel Hartmann

**Affiliations:** a Department of Surgery, TUM School of Medicine, Klinikum rechts der Isar, Technical University of Munich, Germany; b Department of Medicine II, TUM School of Medicine, Klinikum rechts der Isar, Technical University of Munich, Germany; c Department of Mathematics, Technical University of Munich, Germany

**Keywords:** Colorectal cancer, Surgery, Liver metastases, Synchronous resection, Staged resection, Survival

## Abstract

Between 2007 and 2016, 140 consecutive patients who underwent resection of colorectal cancer with simultaneous liver metastases at a single university hospital were retrospectively analysed. In order to gather information regarding potential survival differences for *n* = 68 simultaneous versus *n* = 72 staged resections of the colorectal primary tumor and the liver metastases, Clinical, histopathological, serological, and survival data were compared for those two patient groups. The rate of simultaneous tumor resections increased from approximately 25% in 2007 to >75% in 2016. There was no difference in tumor specific survival for patients with simultaneous vs. staged resection (*p* = 0.631). This effect continued after excluding patients with extrahepatic metastases (*p* = 0.440). Further, neoadjuvant treatment did not lead to differences in the tumor-specific survival (*p* = 0.123). Factors associated with an increased tumor-specific survival were low ASA score (*p* < 0.001), low number of tumor-affected lymph nodes (*p* < 0.001), histological grading G1/2 (*p* = 0.001), and a low number of liver metastases (*p* = 0.044). There was no significant survival difference for the primary tumor stage (pT), the Clavien-Dindo complication rate, the resection status (R0), and minor versus major hepatectomies.

Specifications Table

**Table:** 

Subject	Medicine (Surgery)
Specific subject area	Survival characteristics for patients with synchronous hepatic metastatic colorectal cancer
Type of data	Figure
How data were acquired	Retrospective clinical chart review of patients in the hospital
Data format	Raw
Parameters for data collection	Only data of patients with surgical resection of colorectal cancer and liver metastases were considered. All consecutive patients were included.
Description of data collection	Data collection by review of medical charts, documentation in an Excel data base
Data source location	Supplementary data.
Data accessibility	Supplementary data.
Related research article	U. Nitsche, C. Weber, B. Kaufmann, G. von Figura, V. Assfalg, G. Miller, H. Friess, N. Hüser, D. Hartmann, Simultaneous versus staged resection of colorectal cancer liver metastasis: A retrospective single-center study, J Surg Res. In Press

## Value of the data

• Our data report on a large set of consecutive patients, which is not possible to collect in smaller hospitals. The results help to define treatment algorithms for patients with colorectal cancer and hepatic metastases.
• Abdominal surgeons, oncologists and patients can benefit from these data
• Based on our retrospective analysis, a randomized controlled trial may be conducted in the future regarding the question of simultaneous versus staged resection of colorectal cancer with synchronous liver metastases.
• The data may help to reduced lengths of hospital stays, to reduced costs of the health care system, and to a higher satisfaction of patients.

## Data description

### Experimental design

#### Materials and methods

All medical records of patients who underwent hepatic resections at the Department of Surgery, Klinikum rechts der Isar, Technical University of Munich, Germany, between January 2007 and December 2016, were retrospectively reviewed. Only patients with primary colorectal cancer and synchronous liver metastases were included in this study for further analyses, if a resection of both has been performed, the colorectal primary and the liver metastases, to create a more homogenous study cohort. The decision for simultaneous resection or staged resection was made by evaluation of all available diagnostic findings in the multidisciplinary tumor board, which consisted at least by an oncological HPB surgeon, a gastroenterologist, an oncologist, a radiologist, and a pathologist. The documented data included preoperative performance status; MELD score; tumor staging, including histopathology; details of the surgical and oncological procedures; perioperative morbidity and mortality; liver function tests at different perioperative time points; complication rate according to Clavien-Dindo[2]; and detailed follow-up data. Follow-up was conducted by reviewing the hospital's archiving system or by contacting the patients and their treating physicians. All analyses were conducted on a deidentified data set.

**Fig. 1:**
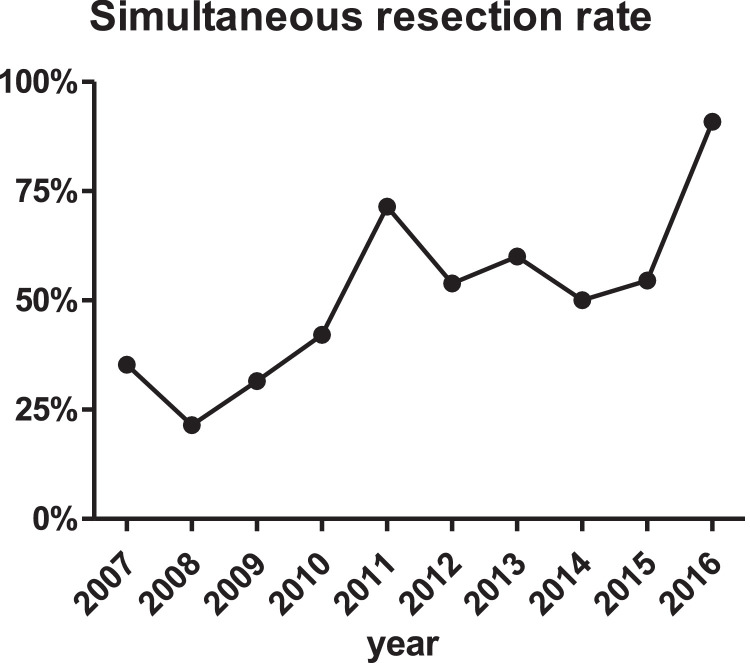
The rate of simultaneous tumor resections (colorectal primary tumor together with liver metastases during one single operation instead of two separate operations) increased from approximately 25% in 2007 to >75% in 2016 (2007: 35%; 2008: 21%; 2009: 32%; 2010: 42%; 2011: 71%; 2012: 54%; 2013: 60%; 2014: 50%; 2015: 55%; 2016: 91%). Clinical characteristics of the patients collective are provided in [1].

**Fig. 2:**
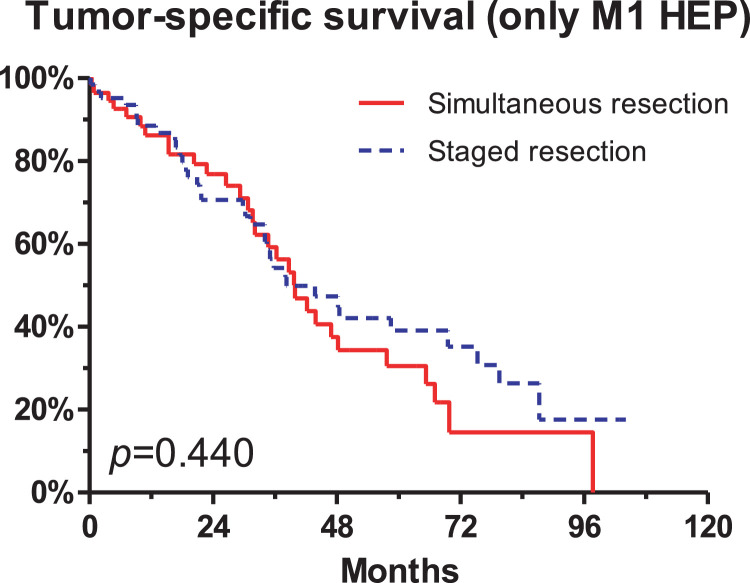
There was no difference in tumor-specific survival in the complete patient cohort, including *n* = 68 patients with simultaneous resection of the colorectal primary tumor and *n* = 72 patients with staged resections (*p* = 0.631; see [1] for the graph and for further description of the whole patient cohort). This finding remained unchanged after exclusion of the *n* = 16 patients who had extrahepatic metastases in addition to the liver metastases (*p* = 0.440).

**Fig. 3:**
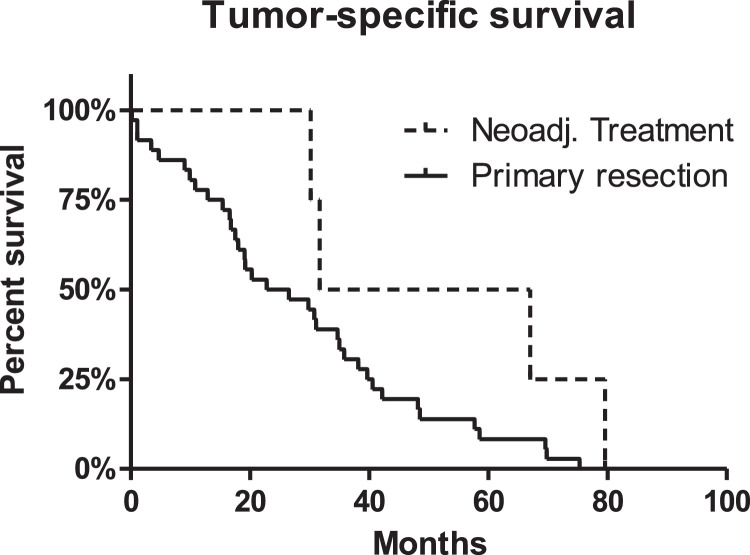
For *n* = 140 patients with hepatic metastatic colorectal cancer, there was no significant improvement of the tumor-specific survival when any kind of neoadjuvant treatment was applied (radiation, chemotherapy, or chemoradiation; *p* = 0.123).

**Fig. 4:**
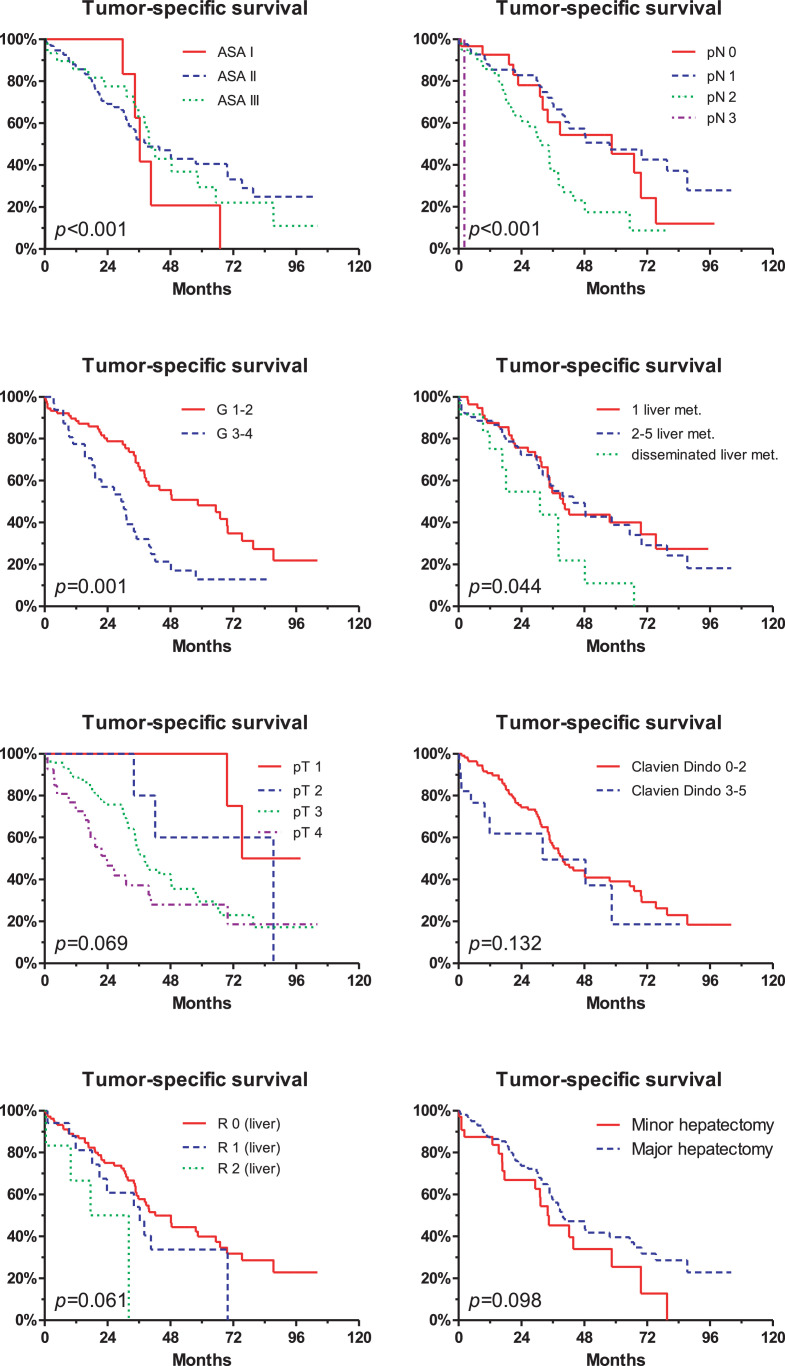
For all *n* = 140 patients, tumor-specific survival differed significantly for patients with different ASA scores (American Society of Anesthesiology), tumor affected lymph nodes, histological tumor grading (G1/2), and number of liver metastases. On the other hand, no survival differences were identified regarding the staging of the colorectal primary tumor (pT), the Clavien-Dindo complication rate [2], the overall resectional status of liver and colorectum (R0), and for minor versus major hepatectomies.

Statistical analyses were performed with SPSS Statistics Version 24.0.0 (IBM Corporation, New York, United States of America) and GraphPad (GraphPad Software, Inc., San Diego, United States of America). Time-dependent survival probabilities were estimated with the Kaplan-Meier method, and the log-rank test was used to compare subgroups.

## Ethics statement

The study was approved by the ethical review board of the Klinikum rechts der Isar of the Technical University of Munich (Ethikkomission an der Technischen Universität München) on March 23th, 2018 (“Datenerhebung von Patienten mit viszeralchirurgischen Erkrankungen”, # 93/18 S). Written, informed consent was obtained from each patient included in the study prior to surgery. The study protocol conforms to the ethical guidelines of the 2000 Declaration of Helsinki as reflected in a priori approval by the institution's human research committee.

## Supplementary Data

The table depicts all *n* = 140 patients, together with the above mentioned analysed parameters as raw data. Each line represents one patient.

## Declaration of Competing Interest

The authors declare that they have no known competing financial interests or personal relationships which have, or could be perceived to have, influenced the work reported in this article.

## Appendix Supplementary materials

Image, application 1
