# Supplementary material for: Survival data on timing of resection of liver metastases in colorectal cancer patients
Source: Data Brief. 2020 Jul 3;31:105973. doi: 10.1016/j.dib.2020.105973 (PMC7341369; doi:10.1016/j.dib.2020.105973)
Supplement: Supplementary file 1 [file mmc1.docx]

**Supplementary data:**

| **Year of liver resection** | **Type of surgery** | **Extrahepatic Metastasis** | **Follow-up time (months)** | **Tumor-related death at follow-up time** | **Any neoadjuvant therapy** | **Any adjuvant therapy** | **ASA Score** | **pT stage (primary tumor)** | **pN stage (affected lymph nodes)** | **Grading** | **Number of liver metastases** | **Clavien Dindo Complication rate**  **for liver surgery** | **Resectional status**  **(R, for colorectum and liver)** | **Extent of liver resection** |
| --- | --- | --- | --- | --- | --- | --- | --- | --- | --- | --- | --- | --- | --- | --- |
| 2007 | simultaneous | no | 32,1 | yes | yes | no | 2 | 3 | 0 | 1-2 | 2-5 | 3-5 | R2 | major |
| 2007 | staged | no | 0,7 | no | no | no | 2 | 4 | 1 | 3-4 | 1 | 1-2 | R0 | minor |
| 2007 | staged | no | 58,5 | no | no | no | 2 | 4 | 1 | 1-2 | 1 | 1-2 | R0 | major |
| 2007 | staged | no | 35,0 | yes | no | no | 2 | 3 | 2 | 3-4 | 2-5 | 1-2 | R0 | major |
| 2007 | staged | no | 1,1 | yes | no | no | 3 | 4 | 2 | 1-2 | 2-5 | 3-5 | R1 | minor |
| 2007 | simultaneous | no | 0,8 | yes | yes | no | 2 | 3 | 0 | 1-2 | 2-5 | 3-5 | R1 | major |
| 2007 | staged | no | 1,2 | no | yes | no | 3 | 3 | 2 | 1-2 | 2-5 | 3-5 | R0 | minor |
| 2007 | staged | no | 87,3 | yes | no | no | 3 | 2 | 1 | 1-2 | 2-5 | 1-2 | R0 | major |
| 2007 | staged | yes | 1,2 | no | no | no | 2 | 3 | 0 | 1-2 | 2-5 | 3-5 | R1 | major |
| 2007 | staged | no | 2,2 | yes | yes | no | 2 | 3 | 3 | 1-2 | 2-5 | 1-2 | R0 | minor |
| 2007 | staged | no | 48,5 | yes | no | no | 3 | 3 | 1 | 1-2 | 2-5 | 3-5 | R0 | major |
| 2008 | staged | no | 75,3 | yes | no | no | 2 | 1 | 0 | 1-2 | 1 | 1-2 | R0 | major |
| 2008 | simultaneous | -missing- | 48,2 | yes | no | no | 2 | 3 | 2 | 3-4 | 2-5 | 1-2 | R0 | major |
| 2008 | staged | no | 102,9 | no | no | no | 2 | 3 | 1 | 1-2 | 2-5 | 1-2 | R0 | major |
| 2008 | staged | yes | 1,1 | yes | no | no | 3 | 4 | 2 | 1-2 | 2-5 | 1-2 | R0 | minor |
| 2008 | staged | no | 79,6 | yes | yes | no | 2 | 3 | 1 | 1-2 | 2-5 | 1-2 | Rx | minor |
| 2008 | staged | no | 21,7 | yes | yes | no | 2 | 4 | 2 | 3-4 | 2-5 | 1-2 | R0 | major |
| 2008 | staged | no | 48,2 | yes | no | no | 2 | 3 | 1 | 1-2 | >5 | 1-2 | R0 | major |
| 2008 | simultaneous | yes | 23,8 | yes | yes | no | 2 | 4 | 2 | 1-2 | 2-5 | 1-2 | R1 | major |
| 2008 | staged | no | 20,9 | yes | no | no | 2 | 3 | 0 | 1-2 | 1 | 1-2 | R1 | major |
| 2008 | staged | -missing- | 9,0 | yes | no | no | 2 | 3 | 2 | 3-4 | 1 | 1-2 | R0 | major |
| 2009 | staged | no | 29,3 | no | yes | no | 2 | 3 | 2 | 1-2 | 2-5 | 1-2 | R0 | major |
| 2009 | staged | no | 0,3 | no | no | no | 3 | 3 | 2 | 1-2 | 2-5 | 3-5 | R0 | major |
| 2009 | staged | no | 21,5 | yes | no | no | 2 | 3 | 1 | 1-2 | 1 | 1-2 | R0 | major |
| 2009 | simultaneous | no | 39,7 | yes | no | no | 3 | 4 | 1 | 1-2 | 2-5 | 1-2 | R0 | major |
| 2009 | simultaneous | no | 38,7 | yes | yes | no | 2 | 3 | 0 | 1-2 | 1 | 1-2 | R0 | major |
| 2009 | staged | no | 69,6 | yes | no | no | 2 | 1 | 0 | 1-2 | 1 | 1-2 | R0 | minor |
| 2009 | staged | no | 58,5 | yes | no | no | 3 | 3 | 0 | 1-2 | 2-5 | 3-5 | R0 | minor |
| 2009 | staged | no | 53,5 | no | no | no | 2 | -missing- | -missing- | -missing- | 1 | 3-5 | R0 | major |
| 2009 | staged | no | 7,0 | no | no | no | 3 | 2 | 0 | 1-2 | 2-5 | 1-2 | R0 | minor |
| 2009 | staged | no | 84,5 | no | no | no | 2 | 2 | 1 | 1-2 | 2-5 | 3-5 | R0 | major |
| 2010 | staged | no | 0,1 | no | yes | no | 2 | 3 | 2 | 1-2 | 2-5 | 3-5 | R0 | minor |
| 2010 | staged | no | 19,1 | yes | no | no | 2 | 3 | 2 | 3-4 | 1 | 1-2 | R0 | major |
| 2010 | simultaneous | no | 9,9 | yes | no | no | 2 | 3 | 1 | 1-2 | 1 | 3-5 | R2 | major |
| 2010 | simultaneous | no | 15,4 | yes | no | no | 2 | 4 | 2 | 3-4 | 2-5 | 1-2 | Rx | minor |
| 2010 | simultaneous | no | 43,9 | no | no | no | 2 | 4 | 2 | 1-2 | 1 | 1-2 | R0 | major |
| 2011 | staged | no | 64,8 | no | no | no | 2 | -missing- | -missing- | -missing- | 2-5 | 1-2 | R1 | major |
| 2011 | simultaneous | no | 17,2 | no | yes | no | 2 | 3 | 0 | 1-2 | 2-5 | 1-2 | R0 | major |
| 2011 | simultaneous | no | 2,3 | no | no | no | 3 | 3 | 2 | 1-2 | 2-5 | 3-5 | Rx | major |
| 2011 | staged | no | 65,0 | no | no | no | 2 | 2 | 1 | 1-2 | 1 | 1-2 | R0 | major |
| 2012 | simultaneous | -missing- | 42,2 | yes | no | no | 3 | 2 | 1 | 3-4 | 1 | 1-2 | R0 | minor |
| 2012 | staged | no | 35,3 | no | no | no | 2 | 3 | 2 | 1-2 | 1 | 1-2 | R0 | minor |
| 2012 | staged | no | 7,1 | yes | yes | no | 2 | 3 | 2 | -missing- | 2-5 | 1-2 | R1 | major |
| 2012 | staged | no | 43,8 | yes | yes | no | 2 | 3 | 2 | 1-2 | 2-5 | 1-2 | Rx | minor |
| 2013 | staged | no | 43,7 | no | no | no | 2 | 3 | 2 | 1-2 | 2-5 | 1-2 | R0 | major |
| 2013 | simultaneous | no | 4,7 | yes | no | no | 3 | 4 | 1 | 1-2 | 2-5 | 3-5 | R0 | major |
| 2013 | staged | no | 41,3 | no | no | no | 3 | 3 | 1 | 1-2 | 2-5 | 1-2 | R1 | major |
| 2013 | simultaneous | no | 0,4 | yes | yes | no | 2 | 3 | 2 | 1-2 | >5 | 3-5 | R2 | major |
| 2014 | staged | no | 31,6 | no | yes | no | 1 | 3 | 0 | 1-2 | 1 | 1-2 | R0 | major |
| 2014 | staged | yes | 26,9 | no | no | no | 3 | 3 | 2 | 1-2 | -missing- | 1-2 | Rx | major |
| 2014 | simultaneous | yes | 25,0 | no | no | no | 3 | 3 | 2 | 1-2 | 1 | 1-2 | Rx | major |
| 2014 | staged | no | 22,5 | no | no | no | 2 | 4 | 2 | 1-2 | 1 | 1-2 | R0 | major |
| 2014 | staged | no | 18,2 | no | yes | no | 2 | 3 | 2 | 1-2 | 2-5 | 3-5 | R0 | major |
| 2014 | simultaneous | no | 24,2 | no | no | no | 2 | 3 | 1 | 1-2 | 1 | 1-2 | R0 | major |
| 2014 | simultaneous | no | 13,5 | no | no | no | 2 | 3 | 0 | 1-2 | 2-5 | 1-2 | R0 | major |
| 2015 | staged | no | 21,3 | no | no | no | 2 | 3 | 2 | 3-4 | 2-5 | 3-5 | R0 | major |
| 2015 | staged | no | 0,2 | yes | no | no | 3 | 3 | 1 | 1-2 | >5 | 3-5 | R0 | minor |
| 2015 | simultaneous | yes | 17,5 | no | no | no | 2 | 3 | 2 | 1-2 | 1 | 1-2 | Rx | major |
| 2015 | staged | no | 13,5 | no | no | no | 2 | 3 | 1 | 1-2 | >5 | 1-2 | R0 | minor |
| 2015 | simultaneous | no | 9,8 | no | no | no | 2 | 3 | 2 | 3-4 | 2-5 | 1-2 | R0 | major |
| 2016 | simultaneous | no | 0,2 | no | yes | no | 2 | 3 | 2 | 1-2 | 2-5 | 3-5 | R0 | major |
| 2007 | staged | no | 29,8 | yes | no | yes | 1 | 3 | 1 | 1-2 | 2-5 | 1-2 | Rx | major |
| 2007 | staged | no | 38,0 | yes | no | yes | 2 | 3 | 2 | 1-2 | >5 | 1-2 | R2 | major |
| 2007 | simultaneous | no | 95,3 | no | yes | yes | 2 | 1 | 1 | 1-2 | 1 | 1-2 | R0 | major |
| 2007 | simultaneous | no | 97,7 | no | yes | yes | 2 | 1 | 0 | 1-2 | 2-5 | 1-2 | R0 | major |
| 2007 | simultaneous | no | 7,2 | yes | yes | yes | 2 | 3 | 2 | 3-4 | 1 | 1-2 | R0 | major |
| 2007 | simultaneous | no | 65,3 | yes | yes | yes | 3 | 3 | 2 | 1-2 | 2-5 | 1-2 | R0 | major |
| 2008 | staged | no | 104,1 | no | no | yes | 3 | 4 | 1 | 1-2 | 2-5 | 1-2 | Rx | major |
| 2008 | simultaneous | yes | 40,6 | yes | no | yes | 1 | 4 | 1 | 3-4 | 1 | 1-2 | R1 | major |
| 2008 | staged | no | 34,0 | yes | yes | yes | 3 | 3 | 0 | 3-4 | 1 | 1-2 | R0 | major |
| 2008 | staged | no | 34,0 | yes | yes | yes | 2 | 2 | 1 | 1-2 | 1 | 1-2 | R1 | minor |
| 2009 | simultaneous | yes | 34,7 | yes | no | yes | 2 | 3 | 2 | 1-2 | 1 | 1-2 | R0 | major |
| 2009 | staged | no | 34,4 | yes | yes | yes | 1 | 3 | 2 | -missing- | 2-5 | 1-2 | R1 | minor |
| 2009 | staged | no | 16,8 | yes | no | yes | 2 | 4 | 2 | 1-2 | >5 | 1-2 | R0 | minor |
| 2009 | staged | -missing- | 87,2 | no | yes | yes | 2 | 3 | 1 | 1-2 | 1 | 1-2 | R0 | major |
| 2009 | simultaneous | no | 26,5 | yes | no | yes | 2 | 4 | 2 | 3-4 | 1 | 1-2 | Rx | major |
| 2009 | simultaneous | no | 39,9 | yes | yes | yes | 3 | 3 | 2 | 3-4 | 1 | 1-2 | R1 | major |
| 2009 | staged | -missing- | 84,8 | no | no | yes | 2 | 3 | 1 | 3-4 | 1 | 1-2 | R0 | major |
| 2009 | simultaneous | no | 31,7 | yes | yes | yes | 2 | 3 | 1 | -missing- | 1 | 1-2 | R0 | major |
| 2009 | staged | no | 38,2 | yes | no | yes | 3 | 3 | 2 | 1-2 | >5 | 1-2 | R0 | major |
| 2010 | staged | yes | 79,4 | no | no | yes | 3 | 3 | 2 | 1-2 | 1 | 1-2 | R0 | major |
| 2010 | staged | no | 12,9 | yes | no | yes | 2 | 3 | 2 | 1-2 | 2-5 | 1-2 | R0 | minor |
| 2010 | staged | no | 35,8 | yes | no | yes | 3 | 3 | 1 | 1-2 | 1 | 1-2 | R0 | major |
| 2010 | simultaneous | no | 57,7 | yes | no | yes | 2 | 3 | 1 | 3-4 | 1 | 1-2 | R0 | major |
| 2010 | staged | no | 71,2 | no | no | yes | 2 | 4 | 1 | 3-4 | 1 | 1-2 | Rx | major |
| 2010 | simultaneous | yes | 3,4 | yes | no | yes | 2 | 4 | 2 | 3-4 | 1 | 1-2 | R2 | major |
| 2010 | staged | no | 54,9 | no | no | yes | 2 | 3 | 0 | 1-2 | 2-5 | 1-2 | R0 | minor |
| 2010 | staged | no | 31,2 | yes | no | yes | 3 | 3 | 2 | 3-4 | 1 | 1-2 | Rx | minor |
| 2010 | staged | no | 9,4 | yes | no | yes | 2 | 3 | 2 | 3-4 | >5 | 1-2 | R1 | major |
| 2010 | staged | no | 18,1 | yes | no | yes | 2 | 3 | 2 | -missing- | >5 | 1-2 | R1 | major |
| 2010 | simultaneous | no | 10,8 | yes | no | yes | 3 | 3 | 1 | 3-4 | 1 | 1-2 | R0 | major |
| 2010 | staged | no | 16,6 | yes | no | yes | 3 | 3 | 2 | -missing- | 2-5 | 1-2 | R0 | minor |
| 2010 | simultaneous | no | 69,8 | yes | no | yes | 2 | 4 | 1 | 1-2 | 2-5 | 1-2 | R1 | major |
| 2010 | simultaneous | no | 67,0 | yes | yes | yes | 1 | 3 | 0 | 1-2 | >5 | 1-2 | R0 | major |
| 2011 | simultaneous | no | 68,2 | no | no | yes | 2 | 3 | 0 | 1-2 | 1 | 1-2 | R1 | major |
| 2011 | simultaneous | no | 67,3 | no | no | yes | 2 | 3 | 1 | 3-4 | 1 | 1-2 | R0 | major |
| 2011 | simultaneous | no | 46,9 | no | no | yes | 3 | 3 | 0 | 1-2 | 1 | 1-2 | R0 | major |
| 2011 | staged | no | 17,5 | yes | no | yes | 2 | 4 | 2 | -missing- | 2-5 | 1-2 | R2 | minor |
| 2011 | simultaneous | no | 29,2 | yes | yes | yes | 2 | 3 | 2 | 3-4 | 1 | 1-2 | R0 | minor |
| 2011 | simultaneous | no | 63,4 | no | no | yes | 2 | 3 | 1 | 1-2 | 2-5 | 1-2 | R0 | minor |
| 2011 | simultaneous | no | 55,8 | no | no | yes | 3 | 3 | 0 | 1-2 | 2-5 | 1-2 | R0 | major |
| 2011 | simultaneous | no | 30,8 | yes | -missing- | yes | 2 | 3 | 1 | 1-2 | 2-5 | 1-2 | R0 | major |
| 2011 | staged | no | 30,2 | yes | yes | yes | 2 | 3 | 2 | 3-4 | 2-5 | 1-2 | R0 | major |
| 2011 | simultaneous | no | 22,8 | yes | no | yes | 3 | 3 | 0 | 1-2 | 2-5 | 1-2 | R0 | major |
| 2012 | simultaneous | no | 36,3 | yes | yes | yes | 1 | 3 | 1 | 1-2 | 2-5 | 1-2 | R1 | major |
| 2012 | staged | no | 18,0 | yes | no | yes | 2 | 3 | 2 | 3-4 | 2-5 | 1-2 | Rx | major |
| 2012 | simultaneous | no | 34,7 | yes | no | yes | 2 | 3 | 2 | 1-2 | 1 | 1-2 | R0 | major |
| 2012 | simultaneous | no | 20,3 | yes | no | yes | 2 | 3 | 2 | 1-2 | 1 | 1-2 | R0 | major |
| 2012 | simultaneous | no | 15,4 | yes | yes | yes | 2 | 3 | 2 | 3-4 | 1 | 1-2 | R0 | major |
| 2012 | simultaneous | no | 50,4 | no | no | yes | 2 | 3 | 2 | 1-2 | 1 | 1-2 | R0 | major |
| 2012 | staged | yes | 43,9 | no | no | yes | 2 | 3 | 2 | 1-2 | 1 | 1-2 | R0 | minor |
| 2012 | simultaneous | yes | 19,2 | yes | no | yes | 2 | 4 | 0 | 3-4 | 1 | 1-2 | R0 | major |
| 2012 | staged | yes | 31,1 | yes | no | yes | 2 | 4 | 0 | 3-4 | >5 | 1-2 | Rx | minor |
| 2013 | simultaneous | yes | 44,9 | no | no | yes | 2 | 4 | 1 | 3-4 | 2-5 | 1-2 | R1 | major |
| 2013 | simultaneous | no | 23,5 | no | yes | yes | 2 | 3 | 2 | 1-2 | 1 | 1-2 | R0 | major |
| 2013 | staged | no | 40,5 | no | yes | yes | 2 | 3 | 1 | 1-2 | 2-5 | 1-2 | R0 | major |
| 2013 | staged | no | 32,3 | no | yes | yes | 2 | 3 | 1 | 1-2 | 2-5 | 1-2 | R0 | minor |
| 2013 | simultaneous | no | 38,3 | no | yes | yes | 2 | 3 | 1 | 1-2 | 2-5 | 1-2 | R0 | minor |
| 2013 | simultaneous | no | 26,4 | no | no | yes | 2 | 3 | 0 | 1-2 | 2-5 | 1-2 | R2 | major |
| 2014 | staged | yes | 29,4 | no | no | yes | 2 | 3 | 2 | 1-2 | 2-5 | 1-2 | Rx | major |
| 2014 | simultaneous | no | 28,4 | no | no | yes | 2 | 3 | 2 | -missing- | 1 | 1-2 | R0 | major |
| 2014 | simultaneous | no | 25,2 | no | no | yes | 3 | 3 | 2 | 1-2 | 1 | 1-2 | R0 | major |
| 2014 | simultaneous | no | 23,2 | no | yes | yes | 2 | 3 | 0 | -missing- | 2-5 | 1-2 | R2 | major |
| 2014 | staged | yes | 11,8 | yes | no | yes | 2 | 4 | 1 | 1-2 | >5 | 3-5 | R1 | major |
| 2015 | simultaneous | no | 3,7 | yes | no | yes | 2 | 4 | 1 | 3-4 | 1 | 1-2 | R0 | major |
| 2015 | simultaneous | no | 16,8 | no | yes | yes | 2 | 3 | 0 | 1-2 | >5 | 3-5 | R0 | major |
| 2015 | staged | no | 13,8 | no | no | yes | 3 | 3 | 1 | 1-2 | 1 | 1-2 | R0 | minor |
| 2015 | simultaneous | no | 9,1 | no | no | yes | 2 | 3 | 2 | 1-2 | 1 | 1-2 | R0 | major |
| 2015 | staged | no | 11,4 | no | no | yes | 2 | 4 | 2 | 1-2 | 1 | 3-5 | R0 | major |
| 2015 | simultaneous | no | 9,7 | no | no | yes | 2 | 3 | 2 | 1-2 | 2-5 | 3-5 | R1 | major |
| 2016 | staged | no | 9,2 | yes | yes | yes | 2 | 4 | 0 | 1-2 | 1 | 1-2 | R0 | major |
| 2016 | simultaneous | no | 6,2 | no | no | yes | 2 | 3 | 2 | 1-2 | 2-5 | 3-5 | R0 | minor |
| 2016 | simultaneous | no | 9,6 | no | yes | yes | 2 | 3 | 0 | 1-2 | 2-5 | 1-2 | R0 | major |
| 2016 | simultaneous | no | 6,3 | no | yes | yes | 2 | 3 | 0 | 1-2 | 2-5 | 3-5 | R0 | minor |
| 2016 | simultaneous | no | 0,3 | no | yes | yes | 2 | 3 | 2 | 1-2 | 1 | 1-2 | Rx | major |
| 2016 | simultaneous | no | 0,7 | no | yes | yes | 3 | 3 | 0 | 3-4 | 1 | 1-2 | R0 | minor |
| 2016 | simultaneous | no | 4,0 | no | no | no | 2 | 4 | 0 | 1-2 | 1 | 1-2 | R0 | major |
| 2016 | simultaneous | no | 1,1 | no | yes | yes | 2 | 3 | 0 | 3-4 | 1 | 1-2 | R0 | minor |
| 2016 | simultaneous | no | 1,4 | no | no | yes | 3 | 4 | 2 | 3-4 | 2-5 | 1-2 | R2 | major |
| 2016 | simultaneous | no | 0,5 | no | no | yes | 2 | 3 | 2 | 3-4 | 1 | 1-2 | R0 | major |
